# Reproducible routes: reliably navigating the connectome to enrich personalized brain stimulation strategies

**DOI:** 10.3389/fnhum.2024.1477049

**Published:** 2024-11-06

**Authors:** Yilin Liu, Mark H. Sundman, Chidi Ugonna, Yu-Chin Allison Chen, Jacob M. Green, Lisbeth G. Haaheim, Hannah M. Siu, Ying-hui Chou

**Affiliations:** ^1^Brain Imaging and TMS Laboratory, Department of Psychology, University of Arizona, Tucson, AZ, United States; ^2^Department of Biomedical Engineering, University of Arizona, Tucson, AZ, United States; ^3^Evelyn F. McKnight Brain Institute, Arizona Center on Aging, BIO5 Institute, University of Arizona, Tucson, AZ, United States

**Keywords:** transcranial magnetic stimulation, personalized brain stimulation, diffusion imaging tractography, resting-state functional connectivity, lateral parietal cortex

## Abstract

Non-invasive brain stimulation (NIBS) technologies, such as repetitive transcranial magnetic stimulation (rTMS), offer significant therapeutic potential for a growing number of neuropsychiatric conditions. Concurrent with the expansion of this field is the swift evolution of rTMS methodologies, including approaches to optimize stimulation site planning. Traditional targeting methods, foundational to early successes in the field and still widely employed today, include using scalp-based heuristics or integrating structural MRI co-registration to align the transcranial magnetic stimulation (TMS) coil with anatomical landmarks. Recent evidence, however, supports refining and personalizing stimulation sites based on the target's structural and/or functional connectivity profile. These connectomic approaches harness the network-wide neuromodulatory effects of rTMS to reach deeper brain structures while also enabling a greater degree of personalization by accounting for heterogenous network topology. In this study, we acquired baseline multimodal magnetic resonance (MRI) at two time points to evaluate the reliability and reproducibility of distinct connectome-based strategies for stimulation site planning. Specifically, we compared the intra-individual difference between the optimal stimulation sites generated at each time point for (1) functional connectivity (FC) guided targets derived from resting-state functional MRI and (2) structural connectivity (SC) guided targets derived from diffusion tensor imaging. Our findings suggest superior reproducibility of SC-guided targets. We emphasize the necessity for further research to validate these findings across diverse patient populations, thereby advancing the personalization of rTMS treatments.

## 1 Introduction

Non-invasive brain stimulation (NIBS) technologies like repetitive transcranial magnetic stimulation (rTMS) hold tremendous therapeutic potential for an expanding list of neuropsychiatric diseases. Since the first Food and Drug Administration (FDA) approval was granted for an rTMS protocol to treat depression in 2008, the agency has cleared therapies for obsessive-compulsive disorder, migraine, smoking cessation, and additional novel protocols for depression (Cohen et al., 2022). Alongside these approvals, research in other areas continues to evolve, yielding promising results for neurodegenerative conditions like Alzheimer's Disease (AD) and other mental illnesses like schizophrenia (Chou et al., 2020; Lorentzen et al., 2022).

Despite remarkable progress in recent decades, there remains considerable opportunity to optimize the efficacy of rTMS therapies, as evidenced by the substantial heterogeneity in therapeutic outcomes (Miron et al., 2021; Fitzgerald et al., 2016). Even for conditions like depression, where rTMS is classified as having “Level A” evidence (i.e., definite efficacy), the treatment can deliver life-changing relief for one subset of patients while an equal number receive no meaningful benefit (Lefaucheur et al., 2020). Given that successful treatment depends upon proper targeting, stimulation site planning represents an aspect of rTMS with outsized potential to optimize treatment outcomes.

For depression, the longest-standing therapeutic application of rTMS, the stimulation target is conventionally determined with scalp-based approaches. The “5- or 6-cm rule,” for example, targets the dorsolateral prefrontal cortex (DLPFC) by moving the coil 5 or 6 cm anterior to the motor hotspot (George et al., 1995). Another scalp-based heuristic adopts the 10–20 system for electroencephalography electrode positioning, where the DLPFC is localized at the F3 location (Zhang et al., 2021). Although these scalp-based techniques have provided an important foundation for rTMS in treating depression, they were not designed to consider the individual variations in head morphology, neuroanatomy, and network topography, which are now recognized as crucial factors for tailoring the stimulation site.

Beyond scalp-based approaches, the next most common practice entails acquiring structural magnetic resonance imaging (MRI), co-registering the image to an anatomical atlas, and positioning the coil over the DLPFC with a 3D neuronavigation system (Rusjan et al., 2010). Findings from studies incorporating structural neuroimaging and neuronavigation systems reveal that conventional scalp-based approaches do not provide precise localization of the DLPFC in 33–68% of patients (George et al., 2010; Herwig et al., 2001). Thus, many postulate that the heterogeneity of rTMS treatment outcomes may be partly due to suboptimal targeting of the DLPFC (Cocchi and Zalesky, 2018; Downar and Daskalakis, 2013; Klooster et al., 2022; Cash et al., 2021c).

Over the last 10 years, a growing body of evidence highlights that the clinical outcomes of brain stimulation are largely influenced, if not dictated, by the **connectivity profile of the targeted stimulation site** (Cash et al., 2021a,b, 2022; Cocchi and Zalesky, 2018; Fox et al., 2014; Klooster et al., 2020; Cardenas et al., 2022; Cole et al., 2020; Rosen et al., 2021). Specifically, in the context of depression, it is increasingly recognized that treatment outcomes are augmented when transcranial magnetic stimulation (TMS) is delivered at sites with stronger negative (i.e., anti-correlated) functional connectivity (FC) with the subgenual anterior cingulate cortex (sgACC). This relationship was elegantly demonstrated by Cash et al., for example, who analyzed data from a study that employed a scalp-based targeting of the left DLFPC in a cohort of patients with depression. Retrospectively, the authors evaluated baseline multimodal MRI to determine the optimal stimulation target in the DLPFC for each individual, dictated by functional connectivity to the sgACC (Cash et al., 2021a). The findings revealed that the average distance between the optimal and clinically implemented stimulation sites was 30 mm, with a strong correlation between the treatment efficacy and the proximity of each individual's optimal target to where rTMS was applied with the scalp-based approach (*r* = −0.60; *p* < 0.001; Cash et al., 2021a). In other words, treatment response was enhanced among individuals who, by chance, received treatment closer to their personalized targets.

Support for the link between therapeutic outcomes and the connectivity profile of the stimulation site is reinforced by research spanning various therapeutic applications and methods of stimulation. Instances of such evidence include retrospective analyses observing that memory enhancement through rTMS was more effective when the locations of experimentally-applied and connectome-guided targets were closely aligned (Cash et al., 2022). Thus, to realize the full clinical potential of rTMS, it may be necessary to employ personalized connectome-guided targeting given the high degree of inter-individual variability in network topology (i.e., connectome fingerprinting; Finn et al., 2015). This personalized, connectome-based stimulation site planning may be particularly necessary for conditions that predominantly affect deeper cortical structures like the hippocampus in AD.

This connectomic approach not only coincides with a recent paradigm shift in modern psychiatry that places a greater emphasis on distributed network disruptions (Braun et al., 2018), but it also aligns with a core feature of rTMS—the ability to induce network-wide neuromodulatory effects (Beynel et al., 2020). While the electromagnetic fields generated by TMS stimuli can only directly stimulate superficial cortical tissue (i.e., depth <30 mm), neural activity can be modulated in deeper brain structures that are functionally and/or structurally connected to the superficial stimulation site (Shafi et al., 2012). Structural and functional connectivity profiles can be evaluated with multimodal MRI to leverage this capability and account for heterogeneous network topology.

The most commonly employed strategy to date utilizes resting-state functional MRI (rsfMRI), which evaluates FC by identifying regions with synchronized fluctuations in spontaneous brain activity at rest. To date, most of the evidence supporting the enhanced efficacy of TMS applied to FC-guided targets comes from retrospective analyses (Cash et al., 2021a, 2022; Rosen et al., 2021; Fox et al., 2014; Weigand et al., 2018). Despite these promising early results, some in the field have highlighted potential limitations associated with evaluations of functional network topography, particularly regarding its reproducibility. Measures of functional network topography exhibit a relatively high degree of intra-individual variability across rsfMRI scans at multiple time points, particularly in older adults (Song et al., 2012; Teeuw et al., 2021; Liu et al., 2023). Consequently, connectivity weights for a given ROI fluctuate over time, destabilizing the FC-guided stimulation target and weakening the reproducibility of this approach (Ning et al., 2019). Another potential limitation that weakens the reliability of this rsfMRI approach is its sensitivity to physiological noise (Birn, 2012). Elbau et al. recently demonstrated how respiratory patterns can significantly influence FC measures and, by extension, interfere with the previously reported relationship between sgACC connectivity to the stimulation site and TMS treatment outcomes (Elbau et al., 2023).

Though less prevalent in the existent literature, SC-guided target selection provides a compelling alternative approach. The structural connectome can be evaluated with diffusion tensor imaging (DTI), which measures the microscopic diffusion of water molecules along axonal pathways to map the projections of white matter (WM) tracts in the brain. The reliability of DTI benefits from the temporal stability of WM tracts, enhancing the reproducibility of SC-guided targets (Momi et al., 2021b). Relatedly, structural network topography is more highly conserved across individuals, producing a tighter clustering of optimal stimulation sites and reducing the risk of coil positioning errors (Luber et al., 2022). With particular relevance for TMS targeting, it is also notable that WM, the underlying physical substrate evaluated by DTI, directly facilitates signal propagation from the superficial target to subcortical ROIs. Early reports from our lab and others demonstrate the feasibility of SC-guided TMS applications. For example, using the hippocampus as the ROI, SC-guided parietal lobe stimulation significantly enhanced memory and hippocampal functional connectivity in a cohort of patients with mild cognitive impairment (Chen et al., 2022).

As the field advances toward tailoring rTMS treatments, assessing the reproducibility between SC- and FC-guided stimulation targets within the same participants is pivotal, laying the essential groundwork for expansive scientific inquiry and clinical trials. Findings of the study would provide critical insight into the consistency and efficiency of targeted rTMS therapy, ultimately contributing to more effective and personalized treatments for neuropsychiatric disorders.

## 2 Methods

### 2.1 Participants

The dataset consisted of 30 right-handed older adults (age: 67.2 ± 7 years; females: 22, education: 16.7 ± 2 years) underwent multimodal MRI scanning at two different but closely spaced time points (mean interval: 16.8 days). The University Institutional Review Board reviewed and approved all protocol procedures.

### 2.2 MRI acquisition and preprocessing

MRI data were acquired by MAGNETOM^®^ Skyra 3 Tesla MRI scanner (Siemens Medical Systems, Erlangen, Germany) with a 32-channel receiver head coil. Foam pads were applied to prevent head motion. The structural MRI protocol included T1-MPRAGE (a 3D gradient echo pulse sequence, T1-weighted) with TR = 2,530 ms, TE = 3.3 ms, TI = 1,100 ms, FA = 7°, FoV = 256 mm, parallel imaging (GRAPPA 2), resolution: 1 × 1 × 1 mm, and T2-FLAIR (a fluid-attenuated inversion recovery MRI sequences, T2-weighted) with TR = 6,700 ms, TE = 101 ms, TI = 2,500 ms, FA = 120°, FoV = 256 mm, parallel imaging (GRAPPA 2), resolution: 1 × 1 × 2.5 mm; scan time = 8 min. Diffusion-weight MRI (single-shot parallel and multi-band dual-spin-echo EPI pulse sequence) parameters with FoV= 256 mm; in-plane matrix size = 128 × 128; in-plane acceleration factor = 2; multi-band factor = 2; TE = 119 ms; TR = 3,700 ms; slice thickness = 2 mm; voxel size = 2 mm^3^; b = 0, 1,000, 2,000, and 3,000 s/mm^2^ as three shell acquisitions for further high angular resolution diffusion imaging (HARDI) approach; number of diffusion-encoding directions = 60; scan time = 9 min. Finally, a resting-state fMRI (rs-fMRI) (T2^*^-weighted gradient-echo EPI pulse sequence, FoV = 240 mm; TR = 3,000 ms; TE = 36 ms; flip angle = 90°; in-plane acquisition matrix size = 160 x 160; voxel size = 1.5 mm^3^; and multi-band factor = 2; scan time = 8 min) was acquired. During the scans, participants were asked to stay awake and hold still, keep their eyes focused on a cross, and allow their thoughts to come and go as they wished.

All MRI data was initially converted to the Brain Imaging Data Structure (BIDS) format using custom scripts. After BIDS conversion, preprocessing was performed on the T1-weighted and rsfMRI data using fMRIPrep v1.0.3 (Esteban et al., 2019). Individual T1 brain image was segmented through FreeSurfer v7.1.1 (https://surfer-nmr-mgh-harvard-edu.ezproxy4.library.arizona.edu/) incorporating the Human Connectome Project's Multimodal Parcellation (HCP-MMP v1.0) atlas. FreeSurfer was applied to both T1 and T2 MRI scans to obtain a more reliable segmentation of the hippocampal subfields with enhanced tissue contrast and landmarks of the internal hippocampal structure.

### 2.3 Functional connectivity-guided strategy

Following the preprocessing of resting-state fMRI data with the fMRIprep, we employed the Nilearn Python package to estimate a seed-to-voxel map. We calculated the seed-based map using the left hippocampal body as the seed and averaged the time series across all voxels within this seed. Physiological noise and other non-neuronal fluctuations were incorporated in the first-level general linear regression model, and the mean signal within the seed mask was subsequently extracted while adjusting for these confounds. A seed-to-voxel connectivity map was generated by positively correlating the seed signal with the signals in each voxel throughout the brain. The residuals from the nuisance regression underwent a bandpass filter between the frequencies of 0.01 and 0.1 Hz. Spatial smoothing of functional data was performed using a 5-mm full-width half-maximum (FWHM) Gaussian kernel.

In the FC-guided approach, we identified superficial stimulation targets within the left lateral parietal cortex (LLPC) by applying a binary parietal mask derived from the HCP-MMP v1.0 Atlas to the connectivity map. This map was further refined by cluster thresholding at the 10th percentile (Cash et al., 2021b), selecting the clusters demonstrating the most robust positive correlation with the left hippocampal body. All surviving clusters were ranked based on the center of gravity (COV), where a higher COV signifies enhanced efficiency in disseminating information throughout the network, potentially indicating a more substantial influence on brain dynamics and the distribution of information. The cluster with the highest COV was selected as the optimal stimulation target for the FC-guided approach.

### 2.4 Structural connectivity-guided strategy

We employed FSL's Bedpostx and Probtrackx tools to create a voxel-wise probabilities tractography map. The diffusion tensor was fitted using the Bayesian Estimation of Diffusion Parameters Obtained using Sampling Techniques (BEDPOSTX) method (Jeurissen et al., 2013). We used probabilistic tractography Probtrackx to reconstruct the distribution probability of tracks (Behrens et al., 2003, 2007). Using this approach, we computed the number of tracts extending from the seed mask (hippocampal body) to the gray matter cortical surface, normalized by the total number of tracts from the seed ROI, thereby generating a probability map from the hippocampal body to the rest of the brain. For this process, we initiated 50,000 streamlines per voxel in the seed mask and produced a probability distribution until they terminated in voxels within the gray matter cortical areas, with a step length of 0.5 mm, curvature threshold of 0.2 and a “loop check” to exclude tracks that double back on themselves. Similar to the FC-guided approach, additional constraint was applied to the probability map by using a binary parietal lobe mask to generate an SC-guided target within the LLPC. The constrained probability map was further cluster thresholded at 10th percentile, and surviving clusters were ranked by their COV. The cluster with the highest COV within the mask was selected as the SC-guided stimulation target.

**Figure 1:**
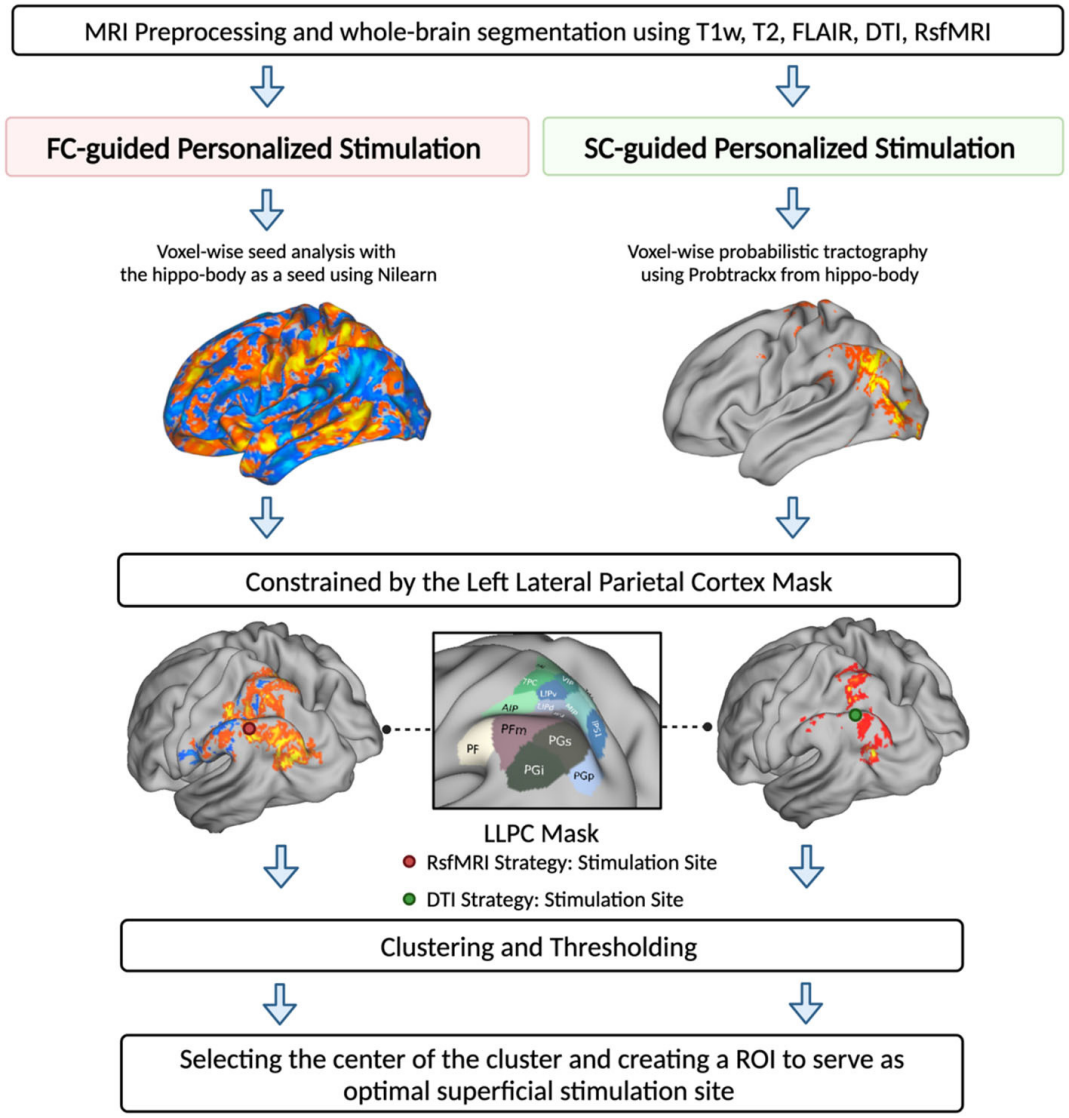
MRI-guided hippocampal TMS targeting strategies. Two targeting strategies were proposed to identify superficial stimulation sites within the left lateral parietal cortex (LLPC). Strategy 1—FC-guided voxel-based approach using seed-based connectivity analysis to identify stimulation sites based on rsfMRI seed-to-voxel connectivity map, centering from the left hippocampal body. Strategy 2—SC-guided voxel-based approach using voxel-wise probability tractography to generate a probability map of tracts extending from the hippocampal body to the cortical surface. For both strategies, we employed a parietal-constrained mask to locate the target within the parietal lobe. After clustering and thresholding, the cluster with the highest center of gravity within each map was chosen as the optimal stimulation site for that strategy.

For both FC- and SC-guided strategies, to make sure that the estimated stimulation sites in the parietal lobe were within the reachable range of TMS (i.e., <30 mm), we utilized the “make_scalp_surfaces” function from an open-source Python package, MNE, to generate head surfaces delineating the interface between the brain, skull, and skin (Gramfort et al., 2013). We then applied the Watershed algorithm from the Boundary Element Method (BEM) to create surfaces for the inner skull, outer skin, and outer skull. We measured the distance between the stimulation site and each voxel in the outer skin to identify the optimal stimulation site that could be reached using TMS (Kozel et al., 2000). A detailed illustration of the optimal stimulation site generation is shown in Figure 1.

### 2.5 Statistical analysis

**Figure 2:**
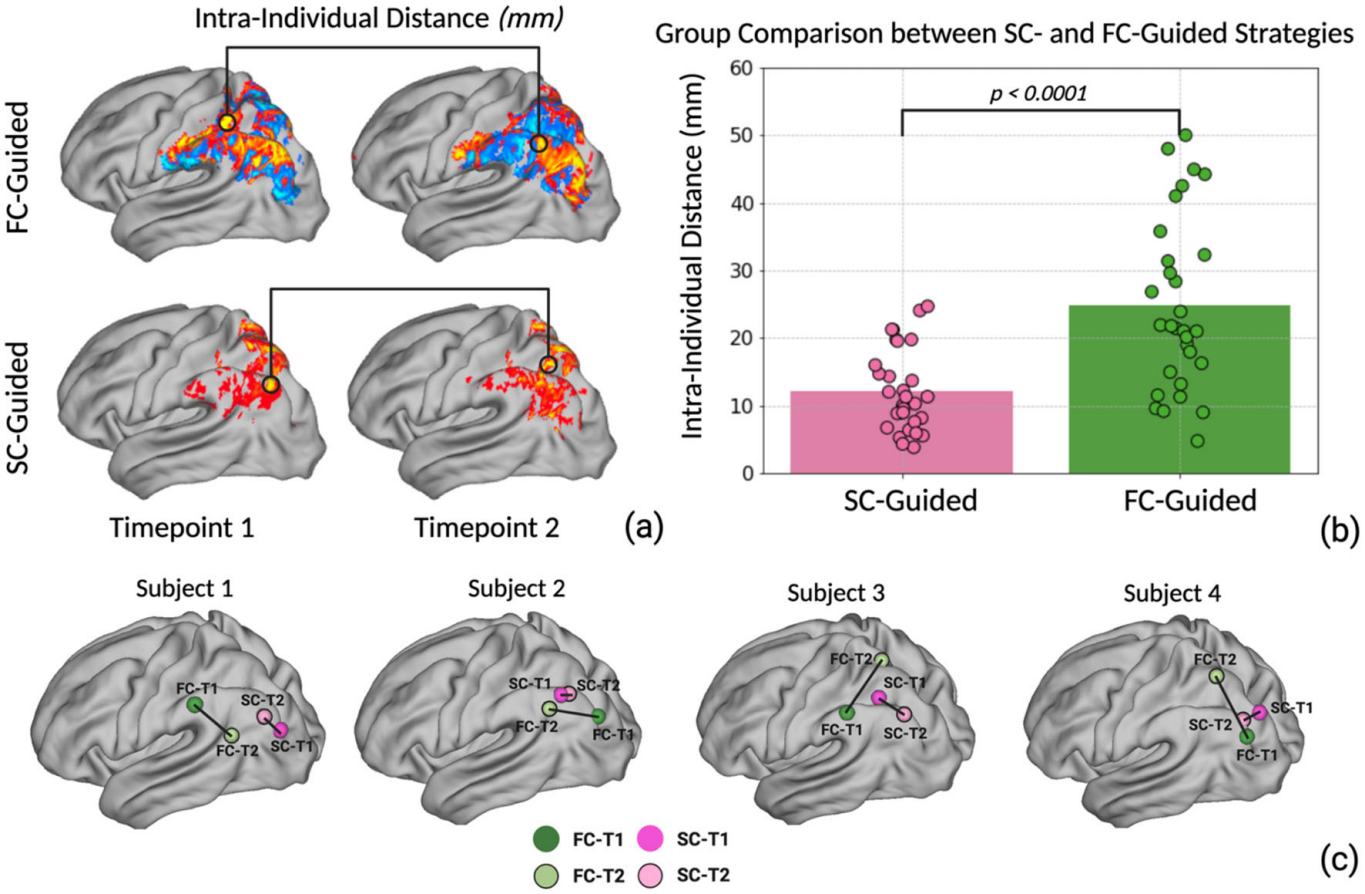
**(a)** The optimal personalized stimulation site coordinates for the SC-guided and FC-guided strategies were computed for 30 individuals at two different time points. The intra-individual distance was calculated based on the Euclidean distance between the two coordinates. **(b)** Welch's *t*-test was performed to investigate the difference between SC-guided intra-individual distance and FC-guided intra-individual distance. **(c)** Illustration of four individual subjects' spatial variation between SC-guided timepoint 1 vs. 2 as well as FC-guided timepoint 1 vs. 2.

To evaluate the reproducibility of the two distinct connectomic targeting strategies, we identified the optimal stimulation site within the left lateral parietal lobe utilizing each strategy at two separate scans (timepoint 1 and timepoint 2). The coordinates of the optimal stimulation targets from each strategy at both time points were subsequently transformed into MNI space for group-level analysis. Next, we evaluated the intra-individual distances between identified stimulation targets generated at each time point by calculating the Euclidean distance between the two coordinates (Figure 2a). Normality of the data distribution was evaluated using the Shapiro–Wilk test for each strategy. To compare the reliability between the two strategies, we employed Welch's *t*-test to analyze differences in intra-individual distance data between two strategies. Welch's *t*-test is robust to violations of normality assumptions and unequal variances between groups. We also calculated the Euclidean distance between the SC-guided and the FC-guided targets at each time point. The average distance between SC- and FC-guided targets is provided in the Supplementary material. Statistical significance was set at *P* < 0.05. All statistical analyses were performed using Python 3.8 with specialized libraries including Scipy, Statsmodels, and Pandas.

## 3 Results

Stimulation targets within the left lateral parietal lobe were produced for every participant using SC-guided and FC-guided methodologies, ensuring the data processing steps remained consistent across both time points (T1 and T2). After transforming coordinates to MNI space, we measured the Euclidean distance between the stimulation sites generated at T1 and T2 for each individual and strategy. The average intra-individual distance between the targets across both time points was 12.2 ± 5.9 mm for the SC-guided approach and 24.75 ± 12.57 mm for the FC-guided approach (Figure 2b). The Welch's *t*-test revealed a significantly smaller intra-individual distance for the SC-guided strategy compared to the FC-guided approach (*t* = −4.856, *p* < 0.0001). This finding suggests a notably higher consistency in pinpointing stimulation targets using the SC-guided approach (Example Illustration Figure 2c).

**Figure 3:**
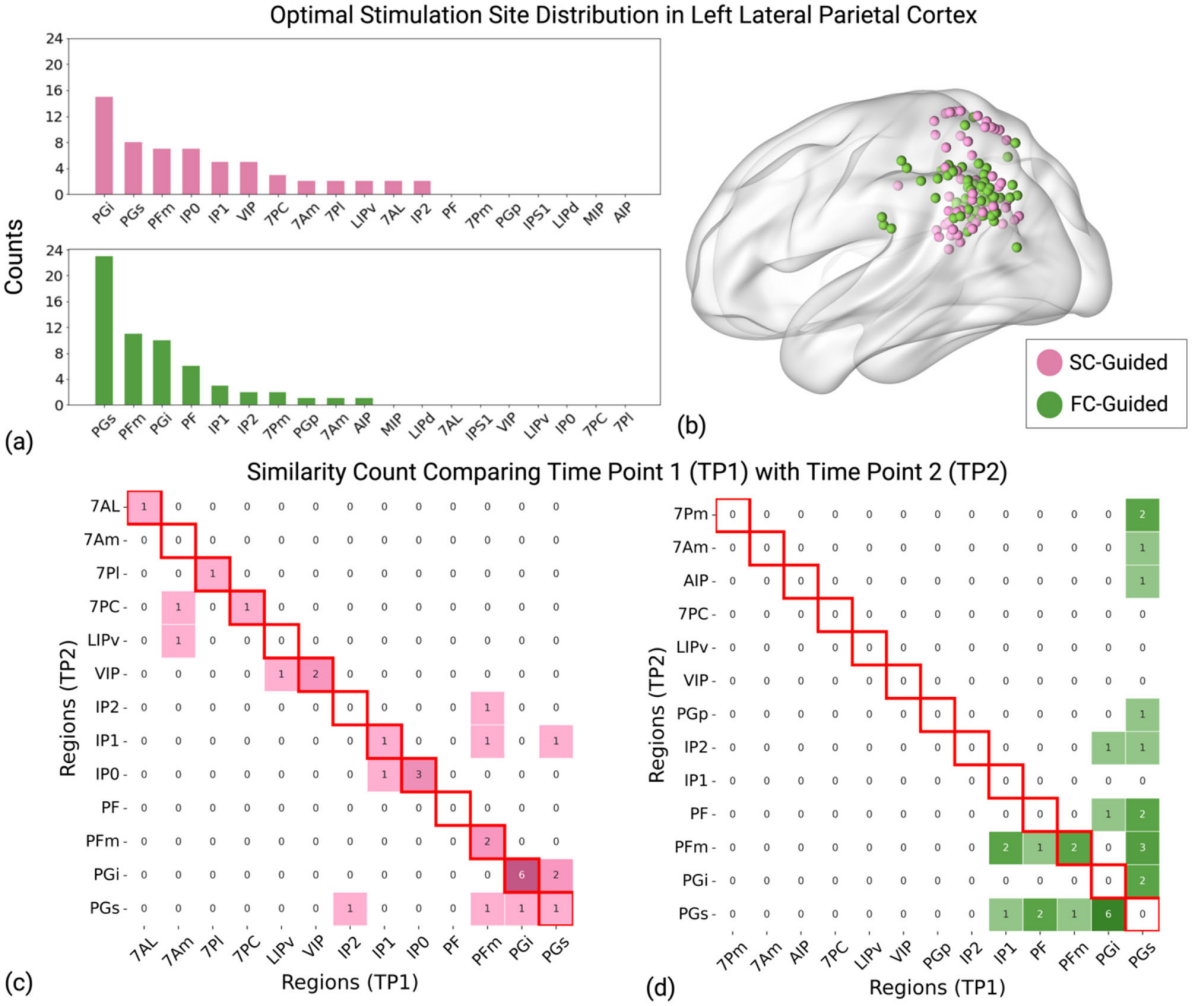
Distribution of identified stimulation sites in the left lateral parietal cortex. **(a)** The y-axis “Counts” indicates the total occurrences of stimulation sites within each strategy (SC-Guided and FC-Guided) across two timepoints in 30 participants (Total 60 occurrences for each strategy). Pink bars denote the SC-guided strategy, and green bars represent the FC-guided strategy. **(b)** Representation of calculated stimulation sites between the two strategies, plotted for all 30 subjects at both timepoints in MNI standard space. **(c)** The matrix represents the count of similarity of identified brain regions between time point 1 (TP1) and time point 2 (TP2) using SC-guided strategy. **(d)** The matrix displays similarity counts for brain regions identified using the FC-guided strategy. Diagonal cells with red borders signify consistent stimulation targets that remain within the same subregion at both time points. A larger number along the diagonal suggests enhanced reliability over time, underscoring the stability of the SC-guided strategy. Pink indicates the SC-Guided strategy, and green indicates the FC-Guided strategy.

Furthermore, we examined how the SC-guided and FC-guided stimulation sites corresponded to the subfields of the left lateral parietal lobe as defined by the HCP-MMP v1.0 atlas and evaluated the consistency of these sites over two time points, T1 and T2 (Figure 3, Supplementary Table 1). The HCP-MMP v1.0 atlas is a detailed map of the human cerebral cortex, covering 180 areas per hemisphere. The HCP-MMP v1.0 atlas integrates data from multiple imaging modalities, including structural MRI for cortical thickness, myelin maps, functional MRI (for both task-based and resting-state connectivity), and topographic gradients in connectivity. This multi-modal approach enables a comprehensive and accurate delineation of cortical areas (Glasser et al., 2016).

The top 5 brain regions identified using the FC-guided approach includes the posterior half of inferior parietal cortex (PGs), the anterior superior surface of the angular gyrus (PFm), the inferior surface of the angular gyrus (PGi), the superior portion of the supramarginal gyrus (PF) and the middle portion of the inferior bank of the intraparietal sulcus (IP1). For further details, interested readers are encouraged to consult Chapter 7: The Lateral Parietal Lobe (Baker et al., 2018). The group average coordinate for the most frequent FC-guided brain region (PGs) was [−42.7, −71.2, 36.4]. For SC-guided stimulation sites, the top 5 brain regions are PGi, PGs, PFm, the posterior portion of the inferior bank of the intraparietal sulcus (IP0) and IP1. The group average coordinate for the most frequent SC-guided brain region (PGi) was [−46.7, −61.6, 26.3]. The mean distance between SC- and FC-guided targets, averaged across both timepoints and participants was 25.58 + 10.82 mm. The distance between SC- and FC-guided targets for each participant is detailed in the Supplementary material.

When evaluating the reproducibility of these targets over time, ~7% of the participants had targets localized to the same LLPC subregions between T1 and T2 for the FC-guided approach. In contrast, roughly 60% of the participants had stimulation targets that remained stable in the same subfields for the SC-guided strategy. This analysis highlights the spatial stability of the SC-guided strategy compared to the FC-guided approach in identifying stimulation sites within the LLPC subregions.

## 4 Discussion

As NIBS therapies continue to evolve by tailoring stimulation sites to each individual's “connectomic fingerprint,” it will be critical to ensure that this personalization is done reliably and reproducibly. In this early work, we demonstrate that the SC-guided strategy for the localization of superficial stimulation sites may be more reliable than the more widely used FC-guided approach. In addition to a significantly smaller intra-individual Euclidean distance between stimulation targets, the reliability of the SC-guided approach is bolstered by the stability of targets remaining in the same subfields of the lateral parietal cortex. Across the two time points, the optimal target was stable within the same subfields for 60% of SC-guided targets compared to just 7% of FC-guided targets.

Beyond the stability of targets within LLPC subregions, our results reveal distinct spatial distributions between FC- and SC-guided targets. FC-guided targets are primarily clustered around the angular gyrus, a lateral hub of the default mode network (DMN) with functional connectivity to the hippocampus. In contrast, SC-guided targets display a bifurcated pattern, with one cluster positioned medially and another slightly inferior to the angular gyrus, showing some overlap. This broader dispersion of SC-guided targets reflects the anatomical constraints of the underlying white matter tracts. In particular, the inferior longitudinal fasciculus (ILF) and cingulum bundle represent major pathways facilitating structural connectivity to the hippocampus, and their divergent projections align with the observed SC-guided target distribution. Notably, the ILF constitutes over 40% of long-range hippocampal projections and is known to extend to the inferior aspect of the angular gyrus (Maller et al., 2019). The enhanced reproducibility of SC-guided targets suggested by our findings is congruent with the broader body of neuroimaging literature. Intra-individual variability in functional network topography is a well-documented limitation of rsfMRI, particularly in aged populations (Teeuw et al., 2021; Song et al., 2012). Conversely, strong test-retest reliability persists for DTI in elderly populations (Luque Laguna et al., 2020; Teeuw et al., 2021). Since our findings are derived from an elderly sample, the instability of FC-guided targets in this study may have been amplified. Future work is needed to determine the generalizability of our findings in younger adults and different patient populations.

In light of this known limitation of rsfMRI data, others have discussed how the implementation of specific rsfMRI acquisition parameters and computational frameworks may improve the reproducibility of FC-guided targets (Cash et al., 2021b). Lengthening the scan duration, for example, can facilitate a more comprehensive and stable assessment of FC by reducing noise, enhancing statistical power, and better capturing the temporal dynamics of brain activity (Finn et al., 2015; Mueller et al., 2015; Birn et al., 2013). Extending these findings to FC-guided TMS targets, Cao and colleagues recently reported that the reliability of FC-guided targets significantly improves when rsfMRI scan times exceed 21 min (Cao et al., 2024). Notably, this is substantially longer than the 8-min rsfMRI scan duration employed in the current study, which is closer to the standard in the field. For context, the scan duration of the DTI scan utilized in this work was 9 min. Future work is required to compare the reproducibility of FC- and SC-guided targets when these rsfMRI parameters are employed.

Though our results demonstrate that SC-guided targeting has enhanced reproducibility, there are also limitations associated with DTI that are worthy of discussion. Fiber tractography aims to delineate the connections between gray matter regions via WM projections to reveal the structural connectome, but interpreting and quantifying DTI data presents challenges (Seguin et al., 2020; Jeurissen et al., 2019). The reconstructed fiber trajectories, derived from microscopic diffusion metrics, are modeled entities that may not directly represent physical nerve fibers. Consequently, concerns arise regarding tractography's limited capacity to trace polysynaptic pathways and account for intersecting fiber crossings (Maier-Hein et al., 2017). Computational methods, like constrained spherical deconvolution (CSD), can mitigate some of these issues by more accurately resolving crossing fibers and modeling WM tracts (Tournier et al., 2012). While limitations around polysynaptic tracing remain, it is notable that connectivity assessed with either DTI or rsfMRI involves polysynaptic connections (Beynel et al., 2020; Momi et al., 2021a). Lastly, neural information flow is inherently directional, and a relevant limitation of DTI in the context of TMS signal propagation is its inability to resolve axonal direction (Seguin et al., 2019).

In light of these limitations, concurrent TMS and functional neuroimaging studies provide evidence that assuages potential concerns regarding the feasibility of SC-guiding TMS targeting. These studies demonstrate that TMS-evoked activity preferentially propagates to regions that are revealed to be structurally connected by tractography modeling (Luber et al., 2022; Momi et al., 2021a,b; Sydnor et al., 2022). Though TMS has been shown to activate functionally connected brain regions, a direct comparison between TMS-evoked activation in functionally and structurally connected regions of the same target site revealed more robust propagation across cortical regions anatomically linked by WM (Momi et al., 2021a). The authors concluded that WM, the substrate of DTI, serves as the “principal conductor for TMS-induced signal propagation” (Momi et al., 2021a). Preferential propagation along WM is also congruent with the notion that direct cortical excitation at the TMS stimulation site primarily occurs in axons, particularly at their terminals or bends (Siebner et al., 2022).

In another elegant demonstration of WM dictating TMS signal propagation, a recent concurrent TMS-fMRI study applied TMS to FC-guided targets in the ventrolateral prefrontal cortex (vlPFC) to modulate amygdalar activity (Sydnor et al., 2022). In a secondary analysis, the authors incorporated DTI tractography to evaluate how structural connectivity influenced evoked responses in the amygdala when TMS was applied to the FC-guided target. Remarkably, the study reported that WM fiber density from the FC-guided target site to the amygdala significantly influenced the magnitude of TMS-evoked activity in this subcortical ROI (Sydnor et al., 2022). In other words, increased WM fiber density corresponds with enhanced neuromodulation in the subcortical ROI, providing empirical support for integrative frameworks that combine SC- and FC-guided approaches to optimize stimulation targets with both structural and functional connectivity to the region of interest (ROI).

While our findings emphasize the critical importance of reliability in personalized rTMS target selection, we acknowledge that their impact and generalizability are limited by the absence of an assessment of the targets' validity. Although SC-guided targets demonstrated greater reproducibility, it remains uncertain whether SC- and FC-guided rTMS interventions are equally effective in achieving the desired physiological and behavioral outcomes. This limitation is readily addressable by future work that is able to incorporate relevant outcome measures to compare the validity of SC- and FC-guided rTMS interventions. Relatedly, this future work could be further augmented by additional investigation into the relative efficacy of novel “consensus” targeting approaches that integrate FC- and SC- profiles of potential stimulation sites. As elegantly demonstrated by Sydnor et al., amygdalar neuromodulation was enhanced when superficial stimulation targets had shared FC- and SC- connectivity to the ROI (Sydnor et al., 2022). Although such inquiry was beyond the scope of the current brief report, we acknowledge the potential benefits of integrating both methods to achieve greater anatomical precision with functional relevance.

By extension, these limitations highlight key unresolved questions regarding hippocampal neuromodulation with rTMS. While our lab and others have provided early evidence of hippocampal neuromodulation through both SC- and FC-guided stimulation of the LLPC, the clinical potential of this approach for memory disorders remains uncertain (Chen et al., 2022; Freedberg et al., 2021). Furthermore, although our results highlight distinct dispersion and stability patterns for stimulation targets with each approach, it remains unknown whether stimulation along specific functional or structural networks is necessary for effective hippocampal modulation. We encourage continued research in this direction to further improve the efficacy of NIBS interventions through more precise and personalized targeting strategies.

## 5 Conclusion

As the evolution of TMS-based therapies continues to accelerate, the outstanding questions are innumerable. Extensive investigation will be required to ascertain the additive therapeutic value of these personalized, connectomic strategies for optimizing treatment responses. Critically, results from this forthcoming research will hinge on the reproducibility of the evaluated connectomic targets. To this end, our results from this early work demonstrate the enhanced reproducibility of SC-guided targets and underscore the need to overtly mitigate the lesser reliability of FC-guided targets.

## Data Availability

The raw data supporting the conclusions of this article will be made available by the authors, without undue reservation.

## References

[B1] Baker C. M. Burks J. D. Briggs R. G. Conner A. K. Glenn C. A. Taylor K. N.. (2018). A connectomic atlas of the human cerebrum-chapter 7: the lateral parietal lobe. Oper. Neurosurg. 15, S295–S349. 10.1093/ons/opy261 30260428 PMC6887702

[B2] Behrens T. E. Berg H. J. Jbabdi S. Rushworth M. F. Woolrich M. W. (2007). Probabilistic diffusion tractography with multiple fibre orientations: what can we gain? Neuroimage 34, 144–155. 10.1016/j.neuroimage.2006.09.018 17070705 PMC7116582

[B3] Behrens T. E. Woolrich M. W. Jenkinson M. Johansen-Berg H. Nunes R. G. Clare S.. (2003). Characterization and propagation of uncertainty in diffusion-weighted MR imaging. Magn. Reson. Med. 50, 1077–1088. 10.1002/mrm.10609 14587019

[B4] Beynel L. Powers J. P. Appelbaum L. G. (2020). Effects of repetitive transcranial magnetic stimulation on resting-state connectivity: a systematic review. Neuroimage 211:116596. 10.1016/j.neuroimage.2020.116596 32014552 PMC7571509

[B5] Birn R. M. (2012). The role of physiological noise in resting-state functional connectivity. Neuroimage 62, 864–870. 10.1016/j.neuroimage.2012.01.016 22245341 PMC13374118

[B6] Birn R. M. Molloy E. K. Patriat R. Parker T. Meier T. B. Kirk G. R.. (2013). The effect of scan length on the reliability of resting-state fMRI connectivity estimates. Neuroimage 83, 550–558. 10.1016/j.neuroimage.2013.05.099 23747458 PMC4104183

[B7] Braun U. Schaefer A. Betzel R. F. Tost H. Meyer-Lindenberg A. Bassett D. S. (2018). From maps to multi-dimensional network mechanisms of mental disorders. Neuron 97, 14–31. 10.1016/j.neuron.2017.11.007 29301099 PMC5757246

[B8] Cao Z. Xiao X. Xie C. Wei L. Yang Y. Zhu C. (2024). Personalized connectivity-based network targeting model of transcranial magnetic stimulation for treatment of psychiatric disorders: computational feasibility and reproducibility. Front. Psychiat. 15:1341908. 10.3389/fpsyt.2024.1341908 38419897 PMC10899497

[B9] Cardenas V. A. Bhat J. V. Horwege A. M. Ehrlich T. J. Lavacot J. Mathalon D. H.. (2022). Anatomical and fMRI-network comparison of multiple DLPFC targeting strategies for repetitive transcranial magnetic stimulation treatment of depression. Brain Stimul. 15, 63–72. 10.1016/j.brs.2021.11.008 34767967 PMC8900427

[B10] Cash R. F. H. Cocchi L. Lv J. Fitzgerald P. B. Zalesky A. (2021a). Functional magnetic resonance imaging-guided personalization of transcranial magnetic stimulation treatment for depression. J. Am. Med. Assoc. Psychiat. 78, 337–339. 10.1001/jamapsychiatry.2020.3794 33237320 PMC7689561

[B11] Cash R. F. H. Cocchi L. Lv J. Wu Y. Fitzgerald P. B. Zalesky A. (2021b). Personalized connectivity-guided DLPFC-TMS for depression: advancing computational feasibility, precision and reproducibility. Hum. Brain Mapp. 42, 4155–4172. 10.1002/hbm.25330 33544411 PMC8357003

[B12] Cash R. F. H. Hendrikse J. Fernando K. B. Thompson S. Suo C. Fornito A.. (2022). Personalized brain stimulation of memory networks. Brain Stimul. 15, 1300–1304. 10.1016/j.brs.2022.09.004 36113762

[B13] Cash R. F. H. Weigand A. Zalesky A. Siddiqi S. H. Downar J. Fitzgerald P. B.. (2021c). Using brain imaging to improve spatial targeting of transcranial magnetic stimulation for depression. Biol. Psychiat. 90, 689–700. 10.1016/j.biopsych.2020.05.033 32800379

[B14] Chen Y. C. Ton That V. Ugonna C. Liu Y. Nadel L. Chou Y. H. (2022). Diffusion MRI-guided theta burst stimulation enhances memory and functional connectivity along the inferior longitudinal fasciculus in mild cognitive impairment. Proc. Natl. Acad. Sci. U. S. A. 119:e2113778119. 10.1073/pnas.2113778119 35594397 PMC9173759

[B15] Chou Y. H. Ton That V. Sundman M. (2020). A systematic review and meta-analysis of rTMS effects on cognitive enhancement in mild cognitive impairment and Alzheimer's disease. Neurobiol. Aging 86, 1–10. 10.1016/j.neurobiolaging.2019.08.020 31783330 PMC6995441

[B16] Cocchi L. Zalesky A. (2018). Personalized transcranial magnetic stimulation in psychiatry. Biol. Psychiat. Cogn. Neurosci. Neuroimag. 3, 731–741. 10.1016/j.bpsc.2018.01.008 29571586

[B17] Cohen S. L. Bikson M. Badran B. W. George M. S. (2022). A visual and narrative timeline of US FDA milestones for Transcranial Magnetic Stimulation (TMS) devices. Brain Stimul. 15, 73–75. 10.1016/j.brs.2021.11.010 34775141 PMC8864803

[B18] Cole E. J. Stimpson K. H. Bentzley B. S. Gulser M. Cherian K. Tischler C.. (2020). Stanford accelerated intelligent neuromodulation therapy for treatment-resistant depression. Am. J. Psychiat. 177, 716–726. 10.1176/appi.ajp.2019.19070720 32252538

[B19] Downar J. Daskalakis Z. J. (2013). New targets for rTMS in depression: a review of convergent evidence. Brain Stimul. 6, 231–240. 10.1016/j.brs.2012.08.006 22975030

[B20] Elbau I. G. Lynch C. J. Downar J. Vila-Rodriguez F. Power J. D. Solomonov N.. (2023). Functional connectivity mapping for rTMS target selection in depression. Am. J. Psychiat. 180, 230–240. 10.1176/appi.ajp.20220306 36855880 PMC11446248

[B21] Esteban O. Markiewicz C. J. Blair R. W. Moodie C. A. Isik A. I. Erramuzpe A.. (2019). fMRIPrep: a robust preprocessing pipeline for functional MRI. Nat. Methods 16, 111–116. 10.1038/s41592-018-0235-4 30532080 PMC6319393

[B22] Finn E. S. Shen X. Scheinost D. Rosenberg M. D. Huang J. Chun M. M.. (2015). Functional connectome fingerprinting: identifying individuals using patterns of brain connectivity. Nat. Neurosci. 18, 1664–1671. 10.1038/nn.4135 26457551 PMC5008686

[B23] Fitzgerald P. B. Hoy K. E. Anderson R. J. Daskalakis Z. J. (2016). A study of the pattern of response to rTMS treatment in depression. Depr. Anxiety 33, 746–753. 10.1002/da.22503 27059158

[B24] Fox M. D. Buckner R. L. Liu H. Chakravarty M. M. Lozano A. M. Pascual-Leone A. (2014). Resting-state networks link invasive and noninvasive brain stimulation across diverse psychiatric and neurological diseases. Proc. Natl. Acad. Sci. U. S. A. 111, E4367–E4375. 10.1073/pnas.1405003111 25267639 PMC4205651

[B25] Freedberg M. Cunningham C. A. Fioriti C. M. Murillo J. Reeves J. A. Taylor P. A.. (2021). Multiple parietal pathways are associated with rTMS-induced hippocampal network enhancement and episodic memory changes. Neuroimage 237:118199. 10.1016/j.neuroimage.2021.118199 34033914 PMC8926059

[B26] George M. S. Lisanby S. H. Avery D. Mcdonald W. M. Durkalski V. Pavlicova M.. (2010). Daily left prefrontal transcranial magnetic stimulation therapy for major depressive disorder: a sham-controlled randomized trial. Arch. Gen. Psychiat. 67, 507–516. 10.1001/archgenpsychiatry.2010.46 20439832

[B27] George M. S. Wassermann E. M. Williams W. A. Callahan A. Ketter T. A. Basser P.. (1995). Daily repetitive transcranial magnetic stimulation (rTMS) improves mood in depression. Neuroreport 6, 1853–1856. 10.1097/00001756-199510020-00008 8547583

[B28] Glasser M. F. Coalson T. S. Robinson E. C. Hacker C. D. Harwell J. Yacoub E.. (2016). A multi-modal parcellation of human cerebral cortex. Nature 536, 171–178. 10.1038/nature18933 27437579 PMC4990127

[B29] Gramfort A. Luessi M. Larson E. Engemann D. A. Strohmeier D. Brodbeck C.. (2013). MEG and EEG data analysis with MNE-Python. Front. Neurosci. 7:267. 10.3389/fnins.2013.00267 24431986 PMC3872725

[B30] Herwig U. Padberg F. Unger J. Spitzer M. Schonfeldt-Lecuona C. (2001). Transcranial magnetic stimulation in therapy studies: examination of the reliability of “standard” coil positioning by neuronavigation. Biol. Psychiat. 50, 58–61. 10.1016/S0006-3223(01)01153-2 11457424

[B31] Jeurissen B. Descoteaux M. Mori S. Leemans A. (2019). Diffusion MRI fiber tractography of the brain. NMR Biomed. 32:e3785. 10.1002/nbm.3785 28945294

[B32] Jeurissen B. Leemans A. Tournier J. D. Jones D. K. Sijbers J. (2013). Investigating the prevalence of complex fiber configurations in white matter tissue with diffusion magnetic resonance imaging. Hum. Brain Mapp. 34, 2747–2766. 10.1002/hbm.22099 22611035 PMC6870534

[B33] Klooster D. C. Vos I. N. Caeyenberghs K. Leemans A. David S. Besseling R. M.. (2020). Indirect frontocingulate structural connectivity predicts clinical response to accelerated rTMS in major depressive disorder. J. Psychiat. Neurosci. 45, 243–252. 10.1503/jpn.190088 31990490 PMC7828925

[B34] Klooster D. C. W. Ferguson M. A. Boon P. Baeken C. (2022). Personalizing repetitive transcranial magnetic stimulation parameters for depression treatment using multimodal neuroimaging. Biol. Psychiat. Cogn. Neurosci. Neuroimag. 7, 536–545. 10.1016/j.bpsc.2021.11.004 34800726

[B35] Kozel F. A. Nahas Z. Debrux C. Molloy M. Lorberbaum J. P. Bohning D.. (2000). How coil-cortex distance relates to age, motor threshold, and antidepressant response to repetitive transcranial magnetic stimulation. J. Neuropsychiat. Clin. Neurosci. 12, 376–384. 10.1176/jnp.12.3.376 10956572

[B36] Lefaucheur J. P. Aleman A. Baeken C. Benninger D. H. Brunelin J. Di Lazzaro V.. (2020). Evidence-based guidelines on the therapeutic use of repetitive transcranial magnetic stimulation (rTMS): an update (2014–2018). Clin. Neurophysiol. 131, 474–528. 10.1016/j.clinph.2019.11.002 31901449

[B37] Liu Y. Lim K. Sundman M. H. Ugonna C. Ton That V. Cowen S. . (2023). Association between responsiveness to transcranial magnetic stimulation and interhemispheric functional connectivity of sensorimotor cortex in older adults. Brain Connect. 13, 39–50. 10.1089/brain.2021.0180 35620910 PMC9942174

[B38] Lorentzen R. Nguyen T. D. Mcgirr A. Hieronymus F. Ostergaard S. D. (2022). The efficacy of transcranial magnetic stimulation (TMS) for negative symptoms in schizophrenia: a systematic review and meta-analysis. Schizophrenia 8:35. 10.1038/s41537-022-00248-6 35853882 PMC9261093

[B39] Luber B. Davis S. W. Deng Z.-D. Murphy D. Martella A. Peterchev A. V.. (2022). Using diffusion tensor imaging to effectively target TMS to deep brain structures. NeuroImage 249:118863. 10.1016/j.neuroimage.2021.118863 34974116 PMC8851689

[B40] Luque Laguna P. A. Combes A. J. E. Streffer J. Einstein S. Timmers M. Williams S. C. R.. (2020). Reproducibility, reliability and variability of FA and MD in the older healthy population: a test-retest multiparametric analysis. Neuroimage Clin. 26:102168. 10.1016/j.nicl.2020.102168 32035272 PMC7011084

[B41] Maier-Hein K. H. Neher P. F. Houde J. C. Cote M. A. Garyfallidis E. Zhong J.. (2017). The challenge of mapping the human connectome based on diffusion tractography. Nat. Commun. 8:1349. 10.1038/s41467-017-01285-x 29116093 PMC5677006

[B42] Maller J. J. Welton T. Middione M. Callaghan F. M. Rosenfeld J. V. Grieve S. M. (2019). Revealing the hippocampal connectome through super-resolution 1150-direction diffusion MRI. Sci. Rep. 9:2418. 10.1038/s41598-018-37905-9 30787303 PMC6382767

[B43] Miron J. P. Jodoin V. D. Lesperance P. Blumberger D. M. (2021). Repetitive transcranial magnetic stimulation for major depressive disorder: basic principles and future directions. Ther. Adv. Psychopharmacol. 11:20451253211042696. 10.1177/20451253211042696 34589203 PMC8474312

[B44] Momi D. Ozdemir R. A. Tadayon E. Boucher P. Di Domenico A. Fasolo M.. (2021a). Perturbation of resting-state network nodes preferentially propagates to structurally rather than functionally connected regions. Sci. Rep. 11:12458. 10.1038/s41598-021-90663-z 34127688 PMC8203778

[B45] Momi D. Ozdemir R. A. Tadayon E. Boucher P. Shafi M. M. Pascual-Leone A.. (2021b). Network-level macroscale structural connectivity predicts propagation of transcranial magnetic stimulation. Neuroimage 229:117698. 10.1016/j.neuroimage.2020.117698 33385561 PMC9094638

[B46] Mueller S. Wang D. Fox M. D. Pan R. Lu J. Li K.. (2015). Reliability correction for functional connectivity: theory and implementation. Hum. Brain Mapp. 36, 4664–4680. 10.1002/hbm.22947 26493163 PMC4803495

[B47] Ning L. Makris N. Camprodon J. A. Rathi Y. (2019). Limits and reproducibility of resting-state functional MRI definition of DLPFC targets for neuromodulation. Brain Stimul. 12, 129–138. 10.1016/j.brs.2018.10.004 30344110 PMC6301130

[B48] Rosen A. C. Bhat J. V. Cardenas V. A. Ehrlich T. J. Horwege A. M. Mathalon D. H.. (2021). Targeting location relates to treatment response in active but not sham rTMS stimulation. Brain Stimul. 14, 703–709. 10.1016/j.brs.2021.04.010 33866020 PMC8884259

[B49] Rusjan P. M. Barr M. S. Farzan F. Arenovich T. Maller J. J. Fitzgerald P. B.. (2010). Optimal transcranial magnetic stimulation coil placement for targeting the dorsolateral prefrontal cortex using novel magnetic resonance image-guided neuronavigation. Hum. Brain Mapp. 31, 1643–1652. 10.1002/hbm.20964 20162598 PMC6871247

[B50] Seguin C. Razi A. Zalesky A. (2019). Inferring neural signalling directionality from undirected structural connectomes. Nat. Commun. 10:4289. 10.1038/s41467-019-12201-w 31537787 PMC6753104

[B51] Seguin C. Tian Y. Zalesky A. (2020). Network communication models improve the behavioral and functional predictive utility of the human structural connectome. Netw. Neurosci. 4, 980–1006. 10.1162/netn_a_00161 33195945 PMC7655041

[B52] Shafi M. M. Westover M. B. Fox M. D. Pascual-Leone A. (2012). Exploration and modulation of brain network interactions with noninvasive brain stimulation in combination with neuroimaging. Eur. J. Neurosci. 35, 805–825. 10.1111/j.1460-9568.2012.08035.x 22429242 PMC3313459

[B53] Siebner H. R. Funke K. Aberra A. S. Antal A. Bestmann S. Chen R.. (2022). Transcranial magnetic stimulation of the brain: what is stimulated? a consensus and critical position paper. Clin. Neurophysiol. 4:22. 10.1016/j.clinph.2022.04.022 35738037 PMC9753778

[B54] Song J. Desphande A. S. Meier T. B. Tudorascu D. L. Vergun S. Nair V. A.. (2012). Age-related differences in test-retest reliability in resting-state brain functional connectivity. PLoS ONE 7:e49847. 10.1371/journal.pone.0049847 23227153 PMC3515585

[B55] Sydnor V. J. Cieslak M. Duprat R. Deluisi J. Flounders M. W. Long H.. (2022). Cortical-subcortical structural connections support transcranial magnetic stimulation engagement of the amygdala. Sci. Adv. 8:eabn5803. 10.1126/sciadv.abn5803 35731882 PMC9217085

[B56] Teeuw J. Hulshoff Pol H. E. Boomsma D. I. Brouwer R. M. (2021). Reliability modelling of resting-state functional connectivity. Neuroimage 231:117842. 10.1016/j.neuroimage.2021.117842 33581291

[B57] Tournier J. D. Calamante F. Connelly A. (2012). MRtrix: diffusion tractography in crossing fiber regions. Int. J. Imag. Syst. Technol. 22, 53–66. 10.1002/ima.22005

[B58] Weigand A. Horn A. Caballero R. Cooke D. Stern A. P. Taylor S. F.. (2018). Prospective validation that subgenual connectivity predicts antidepressant efficacy of transcranial magnetic stimulation sites. Biol. Psychiat. 84, 28–37. 10.1016/j.biopsych.2017.10.028 29274805 PMC6091227

[B59] Zhang M. Wang R. Luo X. Zhang S. Zhong X. Ning Y.. (2021). Repetitive transcranial magnetic stimulation target location methods for depression. Front. Neurosci. 15:695423. 10.3389/fnins.2021.695423 34566561 PMC8458642

