# Supplementary material for: Reproducible routes: reliably navigating the connectome to enrich personalized brain stimulation strategies
Source: Front Hum Neurosci. 2024 Nov 6;18:1477049. doi: 10.3389/fnhum.2024.1477049 (PMC11576443; doi:10.3389/fnhum.2024.1477049)
Supplement: Supplementary file 1 [file Table_1.docx]

**Supplementary Material**

| Distance (mm) between structural connectivity (sc) guided- and functional connectivity (fc)- guided stimulation sites | |
| --- | --- |
| 1 | 17.72 |
| 2 | 34.99 |
| 3 | 40.65 |
| 4 | 23.59 |
| 5 | 48.30 |
| 6 | 36.18 |
| 7 | 11.47 |
| 8 | 16.41 |
| 9 | 27.59 |
| 10 | 21.51 |
| 11 | 23.83 |
| 12 | 18.15 |
| 13 | 26.10 |
| 14 | 17.66 |
| 15 | 13.97 |
| 16 | 10.04 |
| 17 | 16.05 |
| 18 | 16.99 |
| 19 | 22.89 |
| 20 | 18.03 |
| 21 | 12.83 |
| 22 | 25.50 |
| 23 | 41.22 |
| 24 | 48.71 |
| 25 | 20.86 |
| 26 | 21.77 |
| 27 | 26.53 |
| 28 | 30.84 |
| 29 | 36.99 |
| 30 | 40.24 |

Supplementary Table 1. Distances between SC- and FC-guided sites for each subject. This table presents the Euclidean distances (in millimeters) between structural connectivity (SC)-guided and functional connectivity (FC)-guided stimulation targets, calculated at two timepoints for each subject. The values shown represent the average distance across both timepoints for 30 subjects.
